# Saudi Learner Translation Corpus: The design and compilation of an English–Arabic learner translation corpus

**DOI:** 10.1371/journal.pone.0303729

**Published:** 2024-10-23

**Authors:** Maha Al-Harthi, Amal Alsaif, Eman Al-Nafjan, Fatma Alshihri, Mahmoud Saleh

**Affiliations:** 1 Department of Applied Linguistics, College of Languages, Princess Nourah bint Abdulrahman University, Riyadh, Saudi Arabia; 2 College of Computer and Information Sciences, Al-Imam Mohammad Ibn Saud Islamic University, Riyadh, Saudi Arabia; 3 Department of English, King Saud bin Abdulaziz University for Health Sciences, Riyadh, Saudi Arabia; 4 Department of Translation, College of Languages, Princess Nourah bint Abdulrahman University, Riyadh, Saudi Arabia; 5 Department of Translation, College of Languages and Translation, King Saud University, Riyadh, Saudi Arabia; Shanghai International Studies University - Songjiang Campus, CHINA

## Abstract

This article introduces the Saudi Learner Translation Corpus (SauLTC), an innovative multi-version English–Arabic parallel corpus featuring part-of-speech tagging. We describe the corpus parameters and compilation process and explain how textual processing and sentence alignment are conducted. The participants include 366 student translators, 48 instructors, and 23 alignment verifiers. The corpus provides access to two target versions of every ST to allow the detection of the changes in the translation and revision processes from the initial to the final draft. The translations were collected over three years, yielding 5,160,386 tokens. The metadata of 23 sentence alignment verifiers were added to the analysis as a unique variable to investigate individual differences in the manual verification process. This unidirectional corpus can be used to identify student translators’ strategies and errors in translation and analyze the efficacy of instructors’ feedback. Furthermore, it is accessible via an application and a website. It provides translation teachers and researchers with a database that can help develop corpus-based and corpus-driven teaching materials.

## 1. Introduction

Since its inception 20 years ago, learner translation corpus (LTC) research has witnessed huge developments and expansions in terms of scope, design, methods, and aims, affecting translation research and teaching. According to Granger and Lefer [1], LTCs are highly valuable for educational and research purposes. One of the first endeavors used syllabus-driven stratified parallel corpora to address problematic discourses and process items in translation training [2]. Later, parallel translation corpora were developed for research purposes. However, earlier projects were small-scale, and many were not publicly available. More recent ones have been more extensive; some have an online interface, while others have translation error taggers.

The first LTC to include the proper tagging of translation errors was the Multilingual eLearning in Language Engineering (MeLLANGE⁠) project [3], which comprised texts from various fields, such as legal, technical, administrative, and journalistic, translated by student and professional translators. It covered several European languages, including Catalan, English, French, German, Italian, and Spanish, and was intended as a resource to inform translator pedagogy. Other corpora developed for online access include⁠ Polish and English Language Corpora for Research and Applications (PELCRA) [4] and CorTrad in Brazil. CorTrad, a parallel corpus initiated in 1998, was made available online in Tagnin’s study in 2005 [5]; however, it is no longer accessible at the address provided in this article.

Nevertheless, it is the only multi-version Brazilian Portuguese–English corpus comprising three sub-corpora: technical-scientific, literary, and science journalism. The number of translations in CorTrad’s Australian short stories sub-corpora is three times the number of source texts (STs) because the corpus includes three translations of the same ST: version 1 comprises the translation-trainee’s texts, version 2 includes those revised by native speakers, and version 3 presents the final published texts [6]. Unfortunately, this aspect is neglected in most learner translation corpora despite its indispensability in understanding the mediations of the translation process. For example, Popescu-Belis et al. [7] compiled a corpus of English and French translation exam papers, annotated with the professors’ corrections and evaluations, to evaluate machine translation at the University of Geneva.

Several projects have collated student translations in an electronic format; they range from text banks to fully annotated corpora and differ in terms of their aims, composition, annotation methods, and query facilities. However, translation corpora that incorporate Arabic, specifically English–Arabic learner translation corpora, are scarce compared to many other world languages. Emphasizing the importance of LTCs as a resource in translation teaching, Zaki [8] states that “translation programs … produce a wealth of data from students’ translations …[which] could be the basis for building translation learner corpora to act as searchable databases” (p. 35). Our project aims to contribute to this growing area of research by introducing the English–Arabic Saudi Learner Translation Corpus (SauLTC). This resource has potential applications in research and teaching. Such a corpus can be used to investigate the regularities of translated texts, strategies used by student translators, and patterns of the languages involved. Zaki [8] states that Arabic corpora and corpus tools are greatly underutilized in teaching translation. Therefore, our corpus is specifically designed to be used as a pedagogical tool that offers educational features that can enhance students’ translation competence, together with the annotation schemes currently being developed in the second stage of our project [9]. We believe its value derives from the fact that it is the foundation for developing three unique annotation schemes. The first scheme is for the STs, which, besides the POS tagging, integrates many linguistic and translational aspects such as potential translation problems, the linguistic perspectives from which these problems can be described, translation strategies and procedures, possible reasons for translation problems, the implications of mistranslating an ST element, etc. A similar annotation scheme is devised for the TTs, where errors will be annotated based on a newly devised error taxonomy [9]. Incorporating these aspects in SauLTC can help students become more linguistically and culturally sensitive and thus be more likely to be able to produce successful translations.

This article discusses the development, features, and applications of the SauLTC [10], a unidirectional, multi-version parallel LTC hosted and funded by the Deanship of Scientific Research at a public university in Saudi Arabia. It comprises the graduation projects of student translators, some of which were professionally edited and published.

Our motivation to compile and distribute the SauLTC is four-fold. First, freely available English–Arabic parallel corpora are limited. Historically, lexicographers have relied on comparable English–Arabic corpora as a poorer-quality substitute [11]. Alasmri and Kruger [12] argued that “corpus-based research on translated Arabic is scarce” (p. 768). Thus, to address this scarcity, particularly in learner corpus research and corpus-based translation studies, this study focuses on the SauLTC, one of the first freely accessible parallel LTCs in the Arab world.

Second, SauLTC provides two versions of translations: a sub-corpus of unedited draft translations representing the raw output of student translators and the final edited versions. The SauLTC software allows searching within each sub-corpus separately and conducting a parallel search within two or three sub-corpora. The availability of the draft and final submission sub-corpora facilitates the differentiation between linguistic features attributable to the initial translation process and the results of the post-feedback editing process. Abekawa and Kageura’s [13] English-to-Japanese corpus of drafts is one of the earliest compilations of multi-version corpora. The published versions produced by translators at publishing agencies aim to improve machine translation output and establish a system to train inexperienced and volunteer translators. Studies of the draft and final versions of translated texts can contribute to understanding the translation process [14, 15]. Indeed, some mediations occur as the meaning is conveyed from the source to the target language and finally to the edited version of the translation [14, 15]. These mediations can range from “translationese” (a term used to denote the presence of unusual features in a translated text) to common editorial changes that affect lexical diversity and include simplifications and explication. Access to the draft and final submission allows researchers to examine these mediations and trace them to the translation process or post-translation editing. As Bisiada [16] points out, “Corpus studies of translation and multilingual discourse production have so far neglected to use corpora that reflect the production process of a translation” (p. 289). Thus, the SauLTC contributes to facilitating more process-oriented, corpus-based translation studies.

Third, we added a sentential dimension unique to the SauLTC because it allows the query to be filtered according to the alignment verifier. The automated sentential alignment was verified by volunteers who are also professional translators. Many parallel corpora use sentences as the basic alignment unit. Simard and Plamondon [17] discussed the “bitext correspondence problem” and demonstrated that the most logical correspondence occurs at the sentential level (p. 59). This standard led to a demand for manually produced, sentence-aligned corpora to improve machine translation accuracy. Professional manual alignments are the gold standard in training and evaluating automated alignment software [18]. The SauLTC was aligned using Wordfast, a translation software. Subsequently, the alignment was manually evaluated by professional translators whose profiles are included in the metadata. This additional manual dimension can be a reference alignment for machine translation training and evaluation. Additionally, the differences between automatic alignment, manual evaluation, and inter-evaluation variations can be traced back to each verifier and examined.

Fourth, we aimed to make use of the wealth of students’ translation projects in developing a didactic learner corpus that can enhance the learning experience of translation students at our translation department and, at the same time, assist instructors in teaching translation. We decided to limit ourselves at this stage to one translation direction, English to Arabic, and to only these two languages to make the corpus as pedagogically serviceable as possible. The conceptualization of our didactic corpus includes developing unique annotation schemes for the source texts, the target texts, and translation errors [9], which makes our corpus different. The ST annotation scheme is intended to help students identify problematic areas in STs, giving them insight into how the differences between cultures, language systems, and linguistic conventions can create problems for translators. An English–Arabic translation-specific error taxonomy is also being developed for this corpus to be the basis for the error annotation scheme. Developing, testing, and refining these schemes is currently ongoing in collaboration with computer science experts.

We believe that the value of our corpus lies in being multi-version L2 to L1 (English–Arabic) parallel corpus, featuring POS tagging. This design of only one translation direction is motivated by our aim to focus primarily on transfer errors rather than language errors. The annotation schemes, being developed for the second stage of the project, will add to the uniqueness of this project. These schemes are devised to enrich the corpus with linguistic and translational information that translation students typically need to be able to translate more consciously and successfully. Our future research directions include expanding the corpus in terms of language pairs, translation directions, students’ expertise level (beginner, intermediate, advanced), students’ affiliations (from different Saudi universities), and genres and incorporating a comparable sub-corpus.

## 2. Materials and methods

### 2.1 Composition and participants’ profiles

The SauLTC is a corpus comprising three parallel sub-corpora and learners’, instructors’, and alignment verifiers’ profiles. The first corpus is English ST, whereas the second and third corpora include two versions of the translations: the draft translation (Version 1–the student’s first attempt at translating the ST on their own) and the post-feedback final translation (Version 2). Unlike most LTCs, the SauLTC does not contain several translations (by different student translators) of the same ST, as student translators independently select their ST with their instructor’s prior approval. The students can choose the text type they like to translate, but several criteria govern their choice. 1) The text should not be too easy or too difficult. The students are advised to choose books that target average adult readers, not children, teenagers, or highly specialized people. The choice must be approved by the course teachers, who assess the difficulty of the text and usually consult each other to ensure it is suitable for the course objectives. The text should, however, be reasonably challenging, incorporating common translation problems on different levels, which the student must reflect on in a translation preface. The text difficulty level is determined by teachers, based on their personal judgment, relying on simple formal features such as sentence length and syntactic complexity, lexical complexity, and vocabulary. No specific quantitative methods or formulas are used to measure text difficulty. Instead, the teachers rely on the observation of the use of syntactic features such as subordination and embedding and the use of simple/common or complex/uncommon words. The department relies on the considerable experience of the course instructors in supervising and assessing translation projects. 2) The book must be recent and published within the last five years. 3) The student should translate an average of 6000 words throughout the project. If more than one student chooses to translate parts of the same book, no more than 30% of the book should be translated if translation rights have not been obtained from the publisher. Moreover, the end-users of the SauLTC website could only access small portions of each text. Davies [19] practiced this “snippet defense” approach in compiling the Corpus of Contemporary American English. 4) The student and the supervisor must ensure that the book has not been translated previously. A simple online search could ensure this for any published book translation. If no published translation is found, the department graduation project unit is consulted to ensure that a student has not translated the book before. This unit keeps a record of all the translation projects produced by the students in the department.

The compilation of the SauLTC involved three types of participants. Written informed consent was obtained from all participants in this study. The first and primary group was comprised of female undergraduate student translators who produced the translations. They were all Saudi nationals and native Arabic speakers, with a mean age of 21.84 (*SD* = 0.64). They had experience with specialized translation (spanning different areas in humanities, social, and scientific fields), interpreting courses, and translation technology. They were enrolled in a four-year undergraduate translation program focused primarily on written translation and linguistic courses (semantics, linguistics, and stylistics) to increase their awareness of the linguistic aspects relevant to the translation process. The program culminated with the graduation project course and an internship in any translation-related workplace outside the university. The graduation project course required student translators to translate texts that meet the above criteria and complete the translation over ten weeks, working in a real-life setting and submitting a weekly portion to the supervisor, who reads the translation and provides weekly feedback. The students do not translate in controlled conditions or classroom settings; they translate at home using all the resources translators in real-life workplaces or situations would use. They are encouraged to use the technologies they were trained to use in the translation technologies course, including CAT tools and AI-based applications. Every week, the student meets with her supervisor, who thoroughly discusses the translation with her, highlighting the points of strength and aspects that need to be improved. The student explains how she dealt with the translation problems and used her resources. The teacher ensures that the student has handled the translation appropriately and can justify her linguistic choices.

In the eighth or ninth week, the teacher chooses any part of ST that the student has already translated in the past weeks and asks the student to translate it without using any resources. This quiz aims to ensure that the student used her resources competently and efficiently, reflecting a good understanding of the translation process and her ability to justify her choices.

The student translators’ contribution to the current corpus involved their graduation projects during academic years 2015, 2016, and 2017, together with signed consent forms accessible through their SauLTC profiles. Each student translator was required to complete a form containing information on their language background, graduation project grade, and grade point average (GPA). This information was incorporated as metadata variables that could restrict or expand a search query according to a researcher’s requirements.

Learner profiles included three types of metadata:

1. Data concerning the student translator (e.g., demographic information, educational background, translation experience, use of reference material, GPA, and English proficiency);
2. Data concerning the ST (e.g., text type, genre, and word count).
3. Data concerning the target text (TT; e.g., type of task, feedback, revisions, grade, and use of a reference translation memory, machine translation, or terminology database).

The second group of participants were the instructors who provided feedback on the student translators’ drafts and assessed their final submissions. The instructors were women with at least a master’s degree in translation or linguistics and with different employment backgrounds. Some were college faculty, while others were freelancers or loan faculty members from the College of Language and Translation at a public university in Saudi Arabia. Freelancers were recruited on an hourly basis. The instructors’ consent and information were procured before including their student translators’ translations. The instructors’ backgrounds were optional variables that could be controlled in a search query. Instructor metadata (educational background and experience in teaching translation and supervising translation projects) were also accessible and linked to each of the relevant student translators’ profiles.

The third group of participants were the alignment verifiers, who voluntarily double-checked the automatic sentence alignment. Thus, the verifiers were mainly early-career translators and translation postgraduates, who were 24–35-year-old Saudi nationals. They were all qualified translators; a few held postgraduate degrees in translation, and most had some years of professional experience. They earned a certificate of participation and were acknowledged on the SauLTC website’s “About” page. Their metadata enhanced the research capabilities of the SauLTC. The metadata are related to the verifier’s characteristics, such as age, gender, educational background, qualification, and experience in translation. Individual differences can be analyzed for quality and the number of alterations performed on the automatically produced sentence alignments.

### 2.2 Corpus compilation

**Fig 1:**
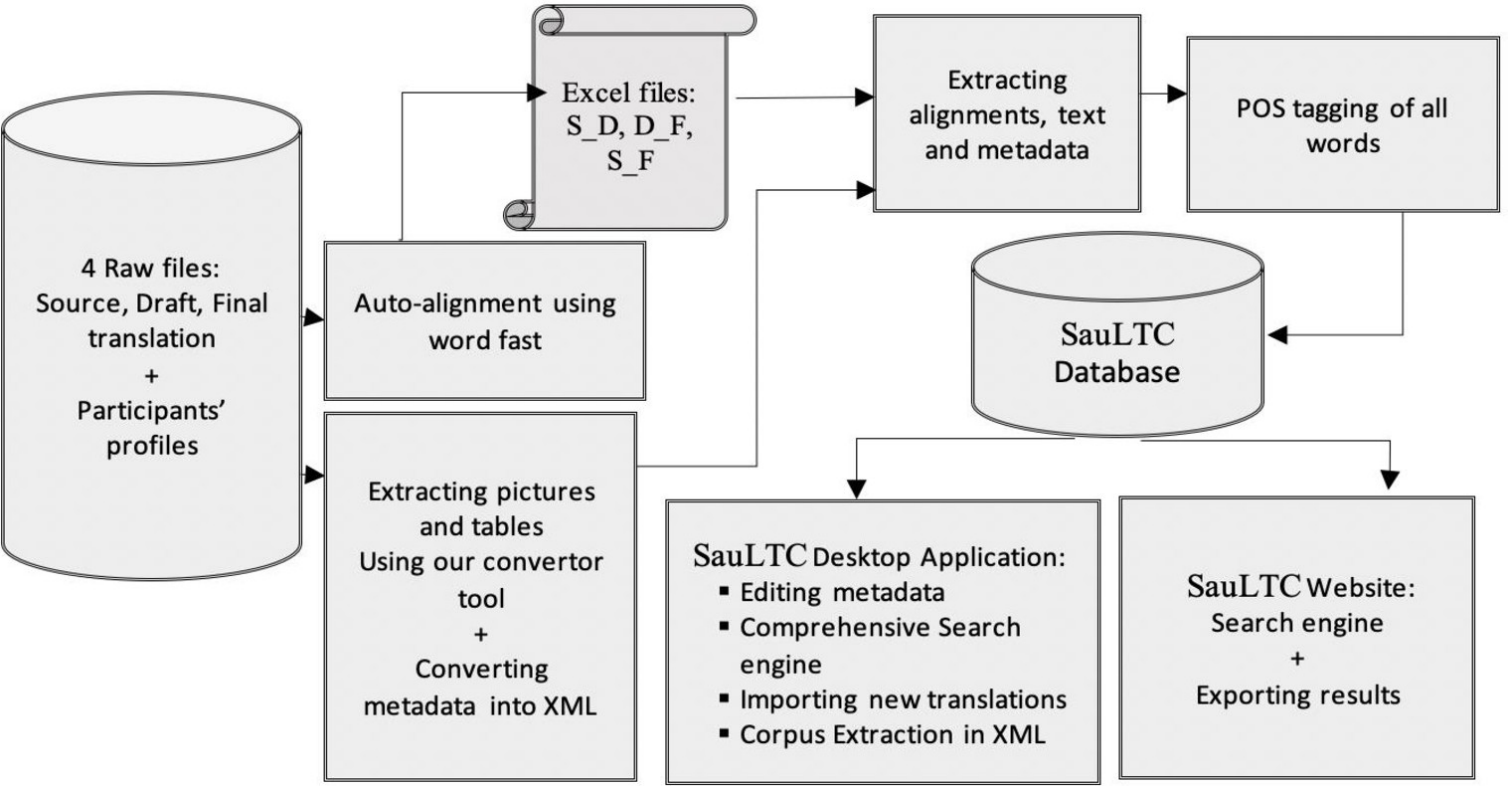
SauLTC framework.

Currently, the SauLTC involves 366 student translators, 48 instructors, and 23 alignment verifiers. Each student’s contribution comprised three files and a learner profile. The three files included the ST, draft translation, and post-instructor-feedback final translation. Table 1 illustrates that the STs were chapters or booklet extracts from various genres and were approximately 6,000 words on average. All three documents were collected in a folder with the students’ names and profiles. Fig 1 illustrates the corpus composition and how the participants’ contributions were fed into two software programs. One was used to extract all the pictures, figures, and tables to be re-inserted later, while the other aligned the sentences among the ST, draft TT, and final TT. Moreover, these processed files were fed into three part-of-speech (POS) taggers and, finally, into the database. Consequently, the output was made accessible on the website and more fully through a specially designed desktop application.

**Table 1:** SauLTC genres.

Genre	Frequency
Health	109
Psychology	41
Self-help	66
Business	51
Parenting	12
Language	12
Religion	1
Education	28
Biography	1
Autobiography	20
Nutrition	11
Management	1
Fiction	7
Social sciences	1
Sciences	3
History	2
Total	366

Table 1 illustrates that student translators’ most popular genre choices were health, self-help, and business, in that order, reflecting their freedom in selecting the texts to translate. The SauLTC search engine allows using genre as a control variable. This parameter can be used to explore the differences between instructors’ approvals and examine whether any of these genres tend to be concentrated among the student translators of one or more of the 48 instructors.

### 2.3 Corpus balancedness

Including texts in the corpus was not based on the researchers’ choice but on practical considerations. As clarified above, the texts were simply translations produced by the final-year students and were chosen by the students and their supervisors without the interference of the researchers. These translations were submitted to the department as part of the requirements of the graduation project course and are considered a wealth of data that we thought could be used as the basis for developing a learner translation corpus. This necessitated adopting an opportunistic corpus-building approach in our project. According to McEnery and Brezina [20], “opportunistic corpus building is sometimes necessary as a response to issues in data collection–for example, copyright restrictions, limited availability of certain types of data or the difficulty of acquiring certain types of data” (p. 275).

Achieving a balanced, proportionate range of genres in a corpus is ideal [20]. However, many researchers have expressed that this principle is sometimes hard to attain, especially when building parallel or translational corpora [21]. Similarly, Doval and Sanchez Nieto [21] contend that this is a challenge that some parallel corpora developers may struggle with or sacrifice depending on their corpora’s nature and objectives. For example, in PEST, a Russian–Finnish–Swedish parallel corpus, the balancedness of genres in one of its sub-corpora (Russian–Finnish) had to be sacrificed because it conflicted with the compilers’ aim to maximize size, which they felt was more important [22]. While compiling their corpus, they found out that the subcorpora were of different sizes because the texts varied in terms of number of texts and word counts. They compensated for this by investing in other aspects such as size, annotation, and the tools users can use to search the corpus [22].

## 3. Results

### 3.1 SauLTC XML Conversion Tool

The first stage involved pre-processing of the raw text. Items were normalized to streamline the alignment process. Illustrations and tables, as well as the student translators’ strategies in handling them, are integral to the translation process; thus, they were included in the SauLTC searchable database. However, automatic sentence aligners can only process running text. Consequently, the diagrams, illustrations, and tables were saved separately and aligned manually.

A major obstacle was the diversity in translation genres and formatting. This lack of uniformity led us to develop the SauLTC XML Conversion Tool. Moreover, we created this tool to extract extra-textual items because there were numerous student folders. The extractions were saved as JPEG files within the respective student translator’s folder under the corresponding sequence identification filenames. The tool was effective but not very accurate in distinguishing in-text illustrations and diagrams from irrelevant paragraph lines, borders, and other embellishments, which must be deleted manually from each student’s folder after extraction. Only then were the relevant diagrams and tables added to the database to be accessed by the researcher as required. However, saving illustrations and tables in a separate file resulted in losing their placement within the text. References to the figures within the texts could assist, but they could not be pinpointed in the original submission. Nevertheless, this was the most efficient way to automatically process the raw text without completely losing the extra-textual elements.

**Fig 2:**
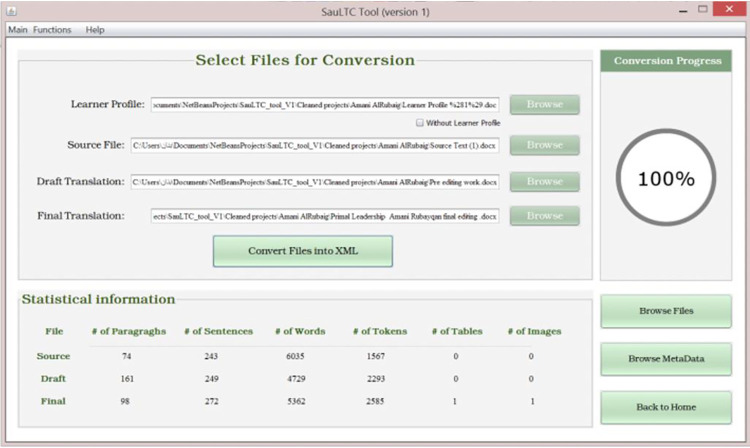
SauLTC XML Conversion Tool V.1 main interface.

**Fig 3:**
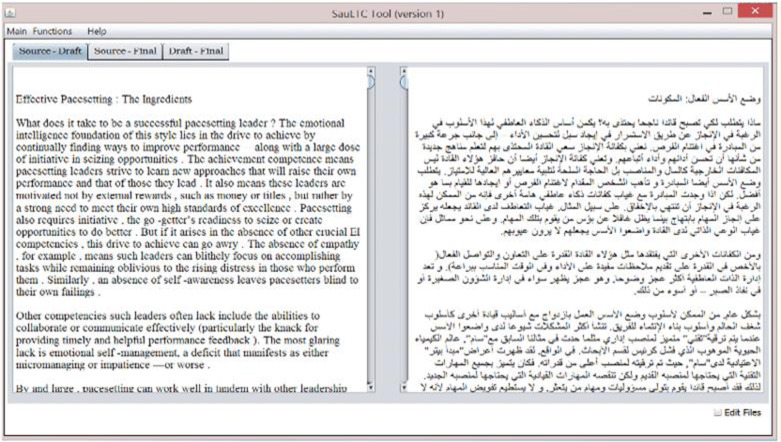
SauLTC XML Conversion Tool V.1 editable interface before conversion.

The tool could convert any MS Word file (English or Arabic) into an XML standard format and extract figures, tables, and extra-textual shapes. Fig 2 illustrates the tool’s main screen, where the user uploads the source, draft, and final translations with the learner’s metadata. First, the tool identified and removed headers, footers, and decorative shapes. After completing the conversion process, statistical information (number of paragraphs, sentences, words, unique tokens, tables, and images) for all three files was displayed. These statistics can be used to check the translation quality and the modifications made by the student in the final version, compared with the draft version. The tool also allowed browsing and editing of the extracted text. The user could modify the text, as illustrated in Fig 3, and save the new edits as XML.

### 3.2 Parallelization and sentence alignment

**Fig 4:**
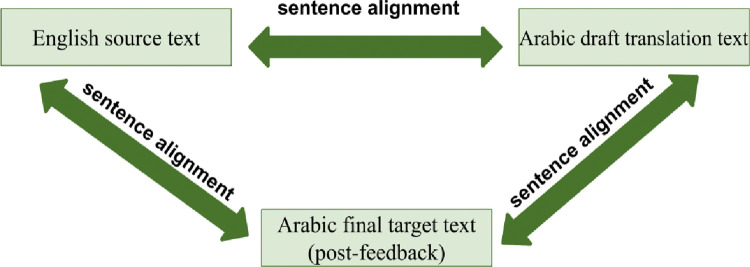
Sentence alignment across the three file types.

The SauLTC is a sentence-aligned bilingual corpus (see Fig 4). The alignment process involved automatic alignment (English–Arabic and Arabic–Arabic pairs) and manual verification.

#### 3.2.1 Automatic alignment

The AutoAligner in Wordfast Anywhere was utilized for the provisional parallelization of the source, draft, and final submission texts. Wordfast Anywhere is a free web-based set of translation memory products; it was selected after carefully testing other applications.

#### 3.2.2 Manual verification

The automated process resulted in some English–Arabic misalignments, where the placement of specific sentences did not correspond with the sentences in the ST. Some ST sentences were missing corresponding translations in the TT, while others had indirect translations included in previous or subsequent TT sentences. Given such instances, manual verification was necessary and was handled by verifiers. They were all graduates or postgraduates with a bachelor’s degree in translation and experience using alignment tools.

**Fig 5:**
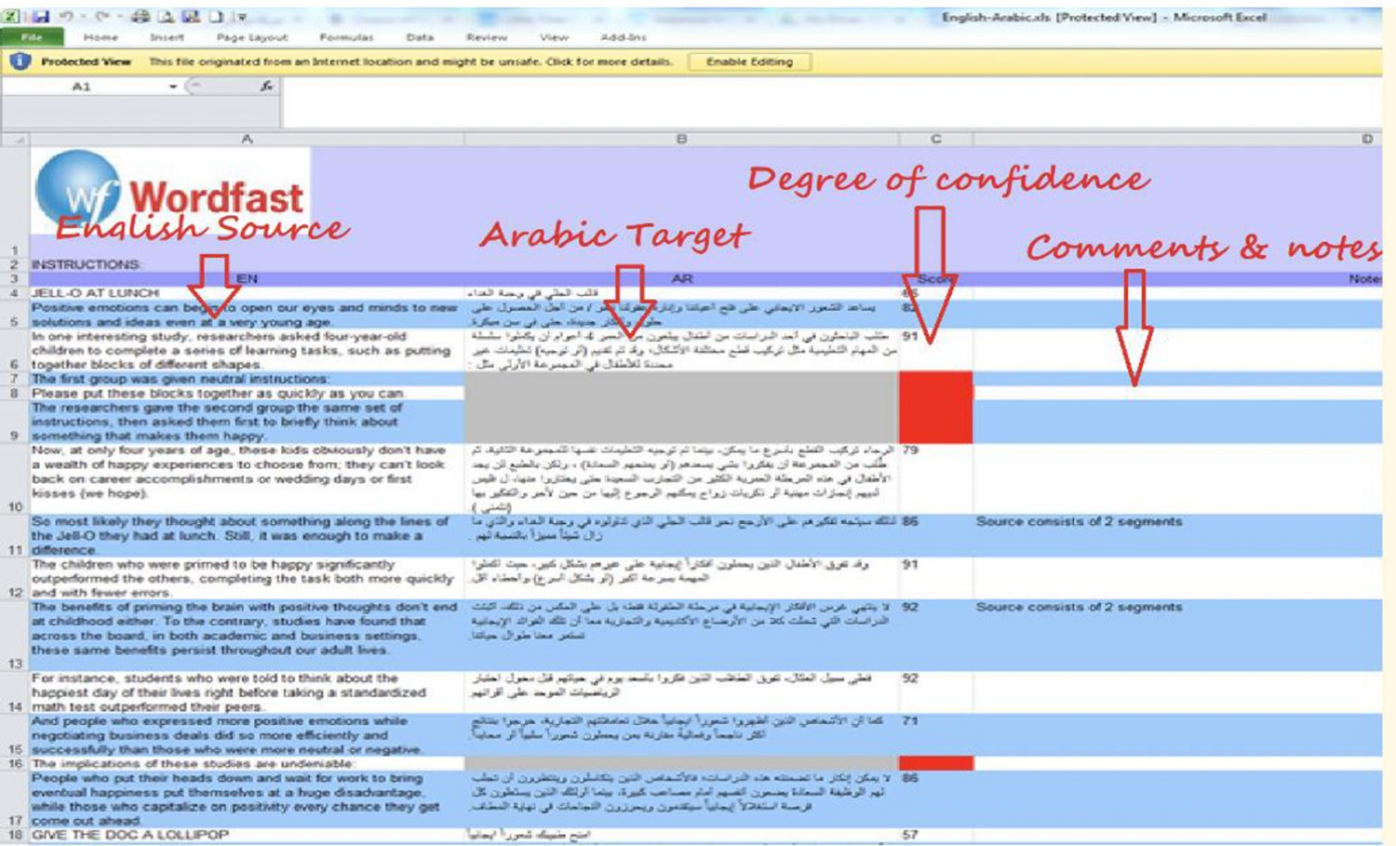
Wordfast sentence aligner Excel output.

Each verifier received at least three student translator folders containing four MS Word files each (source, draft, final translation, and metadata). The verifiers were provided with the SauLTC alignment user manual. We produced the manual and tutorial video to ensure all the details were sequentially explained. Additionally, we provided them access to the SauLTC project team, who could be contacted via email, phone, and in-person meetings for case-by-case support and troubleshooting. The verifiers were required to note any observations, changes, or sentence relocations during the alignment process in a designated column (Fig 5). This commentary can enable future researchers to track translation changes due to the alignment process.

Once the double-check was completed and comments on unusual occurrences were reported, the verifiers filled out a short online form indicating the approximate number of hours taken to complete the verification process, and the number of misalignments found. Subsequently, they emailed three parallelized MS Excel sheets (source_draft, source_final, draft_final) to the SauLTC project team. These Excel sheets were then converted into XML files and used to automatically create the online parallel searchable database using the conversion tool.

The main issue observed by the verifiers was missing punctuation marks, such as full stops. Arabic writers tend to break up long sentences using commas instead of periods in places where the latter would conventionally have been used in English. Furthermore, the automatic sentence aligner relies on periods to identify each unit’s beginning and end, pairing them accordingly. This lack of corresponding punctuation in the target Arabic text lowers the accuracy rate of the automatic parallelization. Wherever a student translator overlooked the need for a period, the software aggregated the running text until it located a period. This sentence convergence resulted in several English source sentences being misaligned with a target Arabic unit.

The missing periods in student translators’ submissions weakened the automatic sentence splitter’s accuracy, as it merged two unpunctuated sentences into one. Thus, we instructed the verifiers to add periods where necessary to safeguard the integrity of the student translations. To minimize data corruption, this was the only punctuation interference allowed for the verifiers. Every added period was reported in a specially designated comments column on the output Excel sheet for further statistical analysis (see Fig 5).

### 3.3 Part-of-speech tagging

The SauLTC was syntactically tagged for Arabic and English. The Stanford Automatic Tagger [23] was used for the English STs, as it is a standard open-source JAVA-based English tagger commonly used in in-text processing systems and research (see Table 2 for the complete list of Stanford POS tags).

**Table 2:** Stanford tagger and MADAMIRA Arabic parts-of-speech (POS) tagsets with SauLTC general mapped tags.

Stanford POS Tag (PTB)	Description	MADAMIRA POS Tag	SauLTC General tag
JJ, JJR, JJS	Adjective	Adj, adj_comp, adj_num	ADJ
RB, RBR, RBS	Adverb	Adv, adv_interrog, adv_rel	ADV
CC, IN	Conjunction–Subordinating	Conj, conj_sub	CONJ
DT EX UH POS UH	Article Determinative Existential there Formulaic Express Genitive Marker Interjection	Part, part_dem, part_det, part_focus, part_fut, part_interrog, part_neg part_restrict, part_verb, part_voc	FUNC
MD	Modal verb	Verb	VERB
(RB)	Negation	NEG	NEG
NN NNS NP, NPS	Noun Common, PluralProper	Noun, abbrev	NOUN
CD CD ORD	Numeral	noun_num, noun_quant, digit	NUM
RP	Particle	Conj	CONJ
IN	Preposition	Prep	PREP
PRP, PRP$, NN, _	Pronoun: Possessive, Nonpersonal Reflexive, Demonstrative	noun_prop, Pron, pron_dem, pron_exclam, pron_interrog, pron_rel	PRONOUN
VB, VB, VBD, VBG, VBZ, VBP, VBN	Verb: Infinitive, Past, Gerund, Pres.part, 3PerSingPres, Non-3PerSingPres, Past Participle	Verb, verb_pseudo	VERB
WDT, WP, WP$, WRB, PDT SENT, TO	WH-determiner, WH-pronoun, Possessive, WH-pro WH-adverb, PreDeterminer Punctuation, To infinitive	-	FUNC
-	Foreign/Latin	Latin	FORENWORD
Punc	Punctuation	Punc	PUNC

MADAMIRA [24] was used to tag the Arabic TTs (see Table 2). The Stanford and MADAMIRA tagsets were not identical; thus, there were discrepancies when the bilingual syntactic classes in the ST and TT were compared simultaneously. To overcome this issue, we created a general tagset (SauLTC General tags) to map these two different tagsets. Some tags had no corresponding equivalent in the other language’s tagset. In these cases, the POS tag in the SauLTC was marked as a No_Gen tag. The SauLTC enabled the end-user to search for a shared tag or tag phrase concurrently within the English source files, Arabic draft, and target files while still using the more language-specific POS tags within the specific language’s text files.

### 3.4 SauLTC application

Upon completing the parallelization process with the manual verification of each Excel alignment file, the files were uploaded to the SauLTC application. This specially designed desktop Java application extracts the sentences for each alignment file, tokenizes the words, and affixes POS tags (Arabic and English) along with the SauLTC General POS tag. We designed a comprehensive database to store all the required information and optimize the corpus search and extraction processes. The main features of the SauLTC application are as follows.

#### 3.4.1 Importing additional translation files

This feature helped expand the corpus. Adding to the corpus required the end-user to provide Wordfast-produced and manually verified Excel files and a corresponding folder with cleaned-out images and table files. The end-user then assigned translation learner and verifier names from a predefined list. If the names were unlisted, the user could add new names by editing the “participants” tab. The text in all the files was extracted into the database, further segmented into words, and saved with the automatic POS tag using Stanford for English and MADAMIRA for Arabic, and the SauLTC General Tagset. Any other figures and illustrations were also saved in the database.

#### 3.4.2 Participants’ profile editing

This feature allowed users to add, delete, or edit the participants’ metadata. The scope of the feature included student translators, translation instructors, and alignment verifiers. The end-user could edit data such as demographic information, educational background, translation experience, and the use of reference material.

#### 3.4.3 Corpus exportation

**Fig 6:**
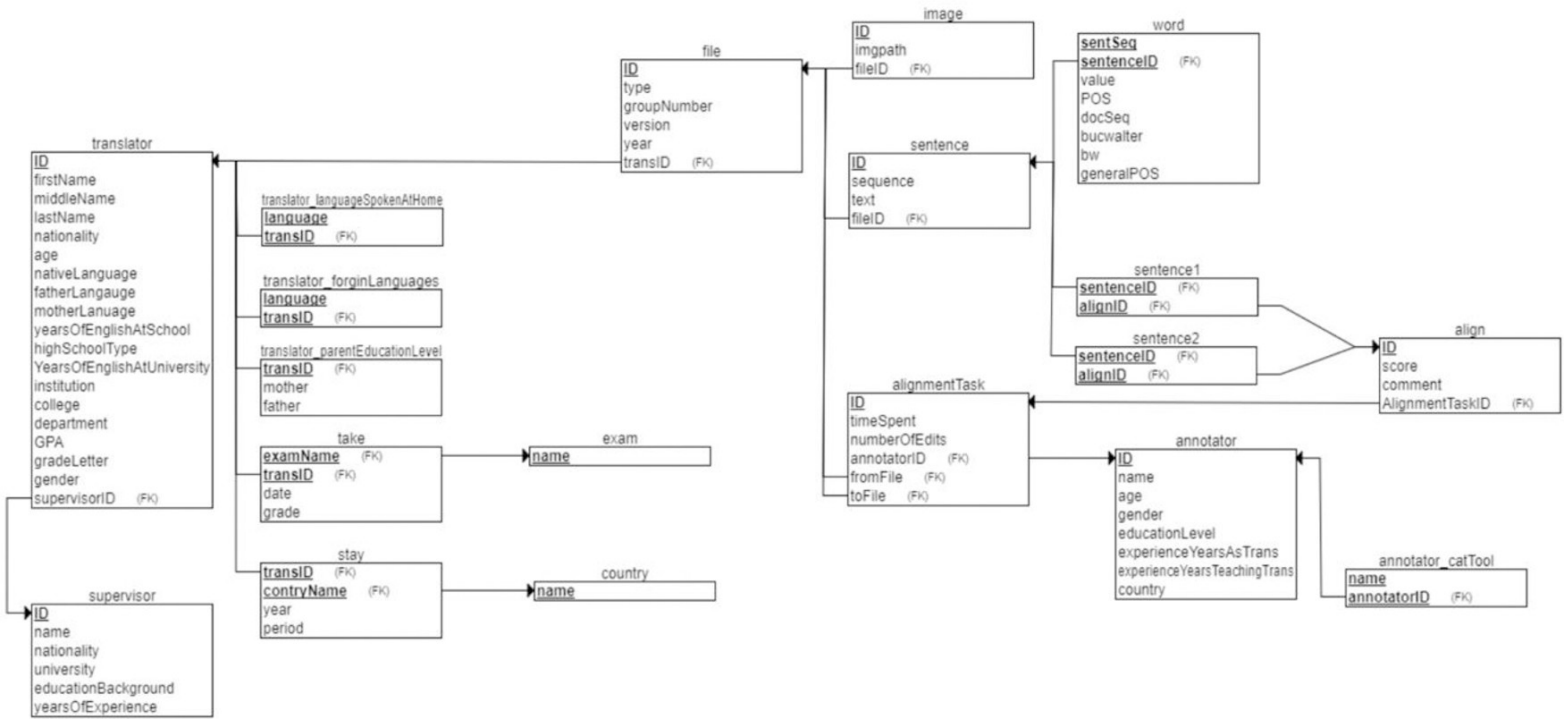
SauLTC database schema (ER-diagram) for the application and website.

This feature allowed extracting the whole corpus or selecting files belonging to a specific verifier, instructor, or learner. Each student translator folder comprised seven XML files: the tokenized text with POS tags and SauLTC General tags of the source, the draft and final translation, participant metadata, source–draft (English–Arabic), source–final (English–Arabic), and draft–final (Arabic–Arabic) alignment files. All these files shared the same naming convention and folder name (i.e., SauLTC_V1_0008_2016_S2; see Fig 6).

#### 3.4.4 Exploring the corpus

**Fig 7:**
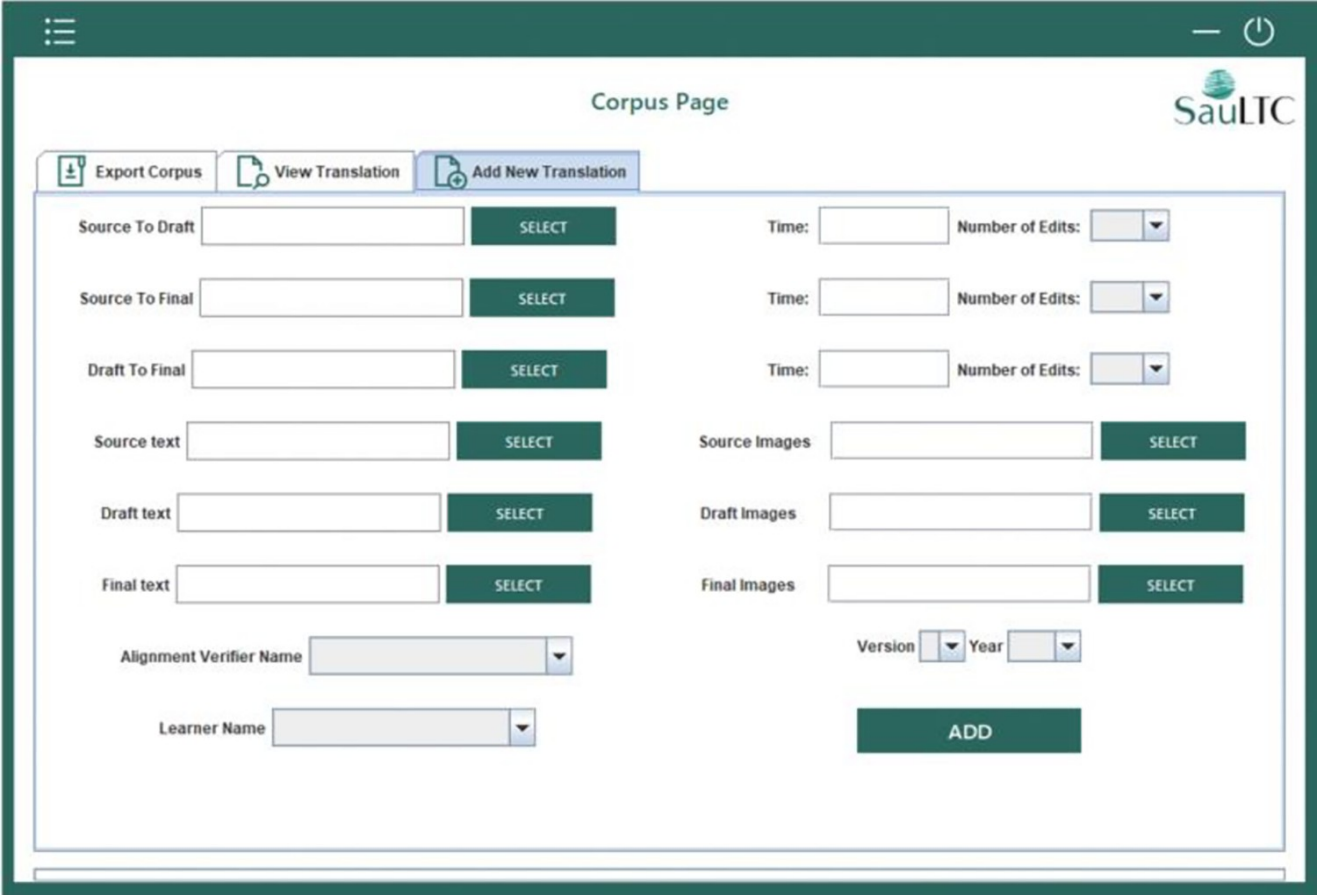
Screenshot of SauLTC translation importation.

**Fig 8:**
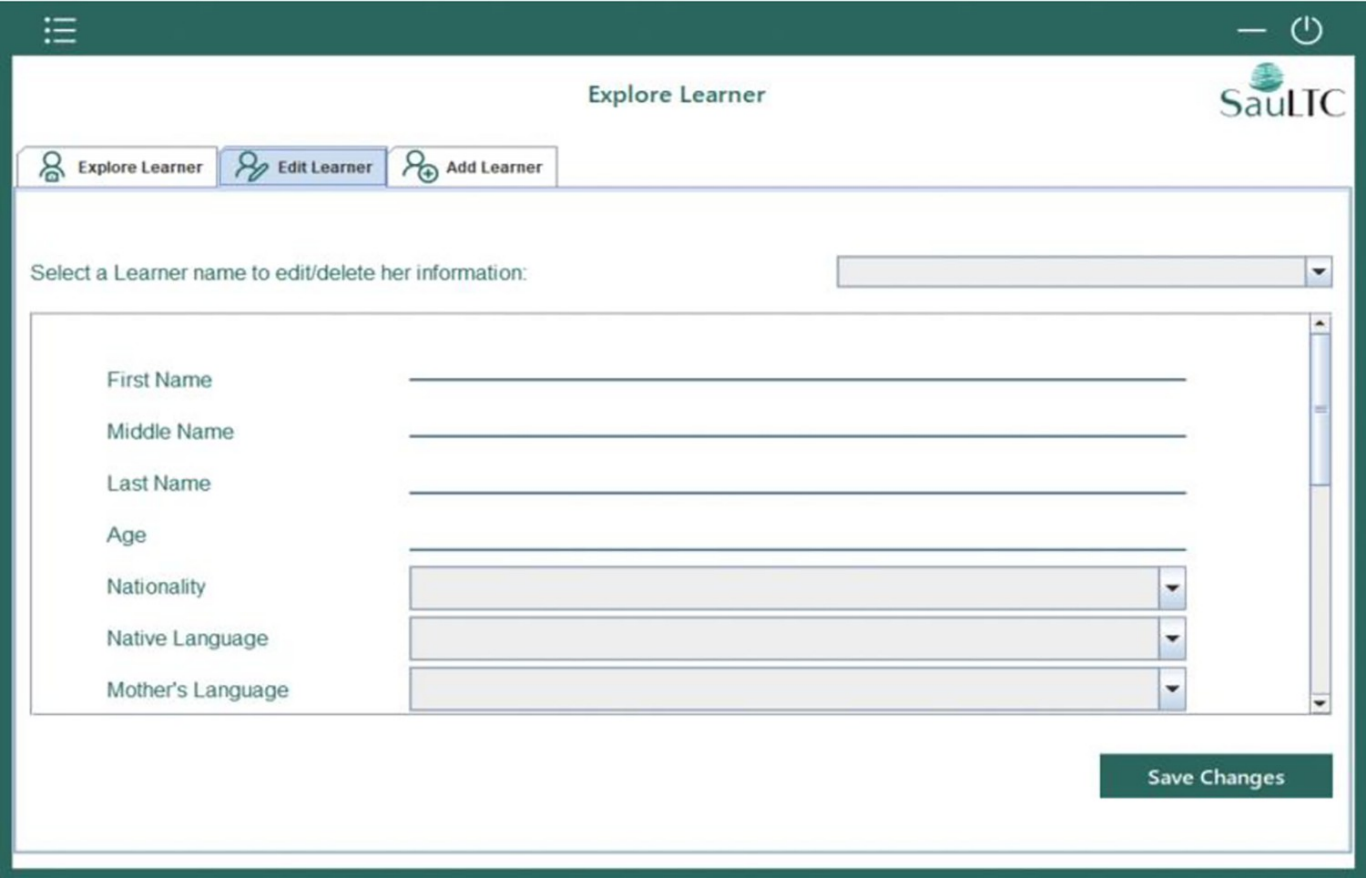
Screenshot of SauLTC application participants’ profile editing.

**Fig 9:**
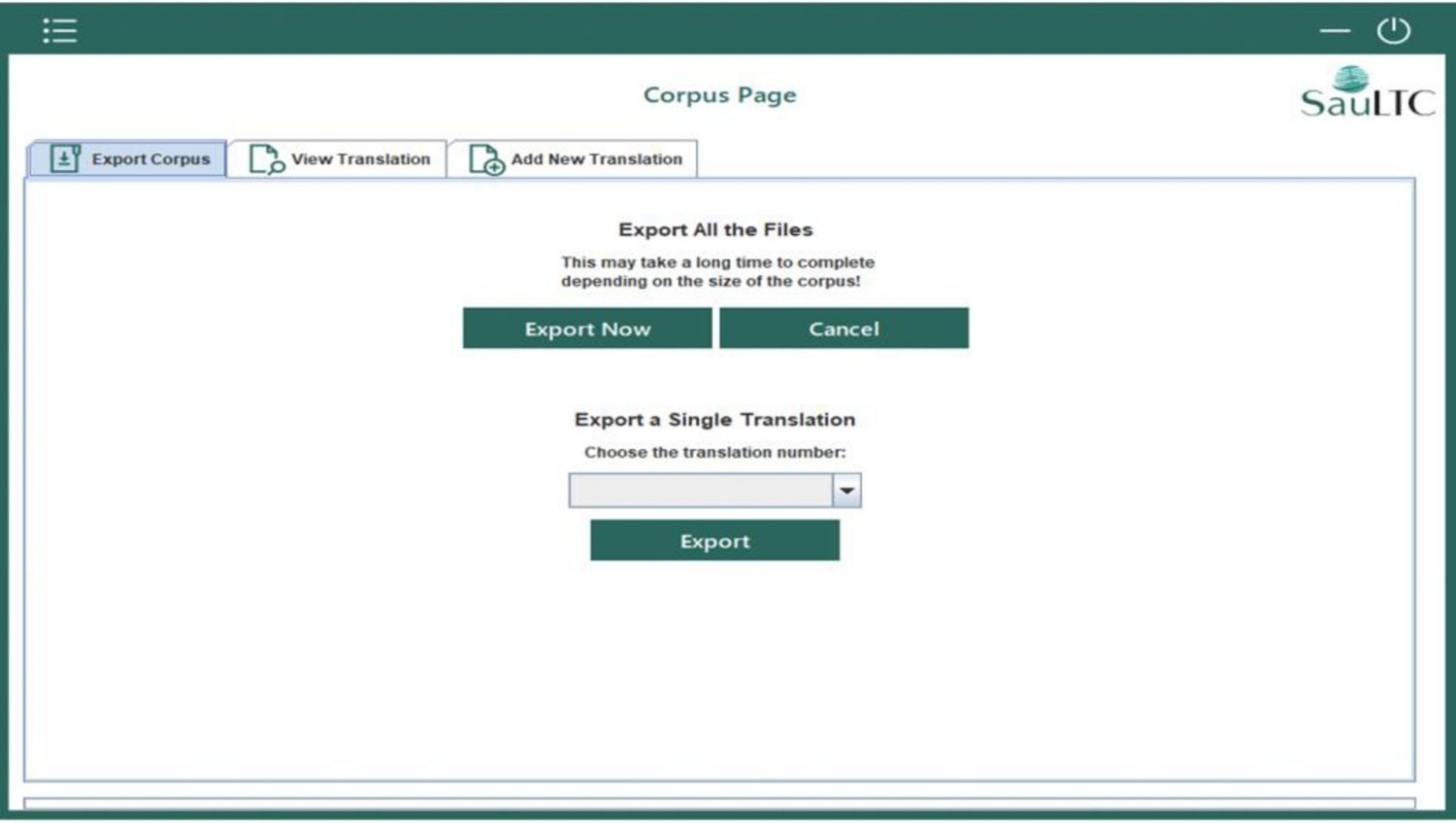
SauLTC application exportation screenshot.

**Fig 10:**
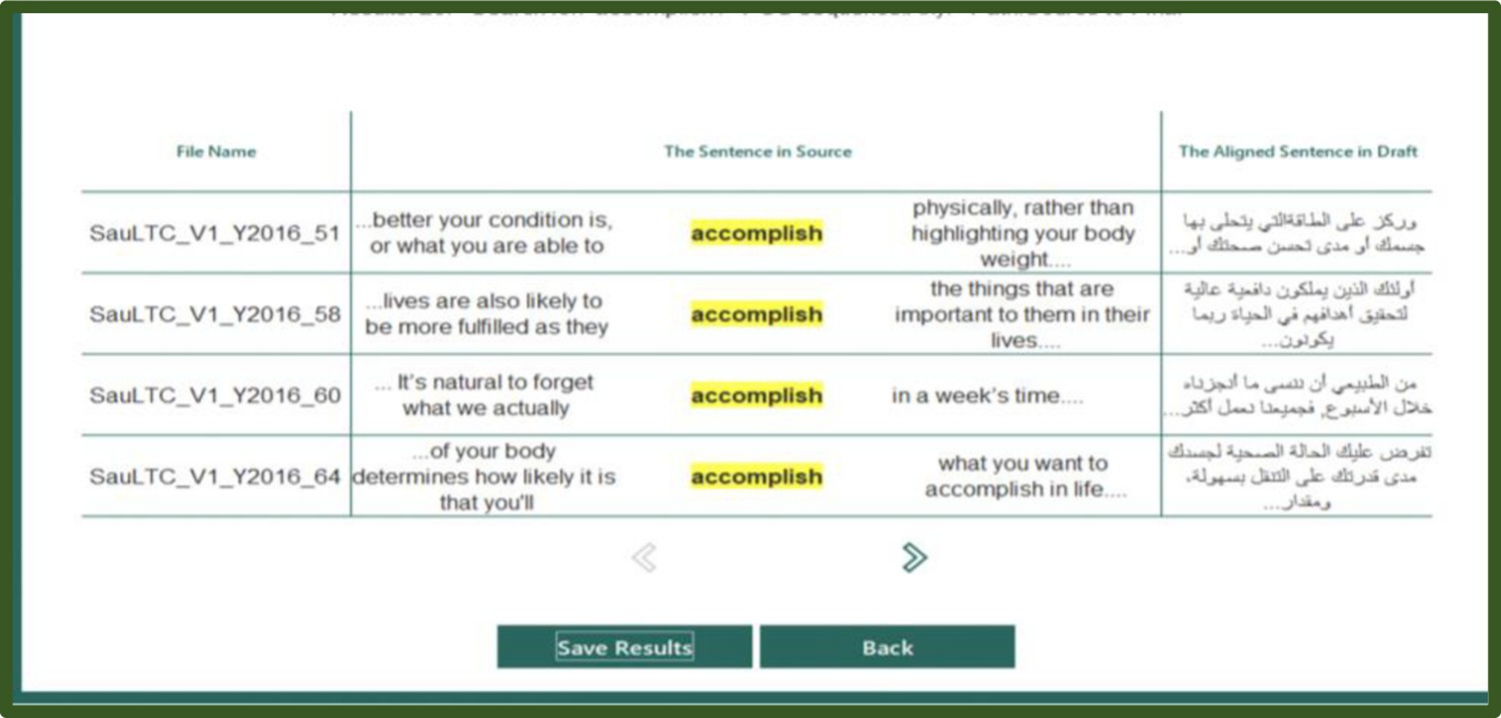
SauLTC application search results.

The most powerful feature of the application is its search capabilities. The user can search simultaneously using single or multiple criteria (see Figs 7–10). First, the user must select the alignment path (source–draft, draft–final, source–final) and enter a word or phrase into the search engine. They could specify the POS tags at either the language-specific level or using the SauLTC tags. Additionally, searches can be conducted using parameters from the participants’ metadata. Advanced searches can be performed with conventional expressions, such as asterisk for wildcard searches and quotations for an exact phrase. Finally, the results of all searches can be downloaded in a CVS file format.

### 3.5 Corpus size

The current SauLTC corpus of 366 translations has 122,417 sentences in the source sub-corpus, 97,456 in the draft sub-corpus, and 97,805 in the final submission sub-corpus. The total number of tokens is 5,160,386, compiled over three years.

More importantly, while there were 122,417 English sentences in the ST files, only 97,456 were aligned sentences in the source–draft alignment files, indicating limits to the one-to-one sentence parallelization in the student translators’ translations. Approximately 224,224 English tokens were omitted when translated into the Arabic draft (1,619,661), and they decreased further to 147,045 words post-feedback in the final submission sub-corpus. These reductions in word count may be explained by the Arabic morphological structure, where a complete and meaningful sentence can be constructed in one token, such as سنكتبها (“we will write it down”). The reduction in tokens from the source sub-corpus to the final sub-corpus was 23%.

For a more detailed examination, we performed an overall count of the POS with the specially designed SauLTC General Tagger (see section 5). The average number of each POS in each sub-corpus is presented in Table 3.

**Table 3:** Parts-of-speech averages across the SauLTC corpus.

	Noun	Prep	Adj	Verb	Adv	Pronoun	Total
Source	1,327	654	471	1,057	311	403.78142	5,582
Draft	1,887	510	429	591	46.8	260.48361	4,278
Final	1,907	514	432	599	46.5	264.54372	4,313

**Fig 11:**
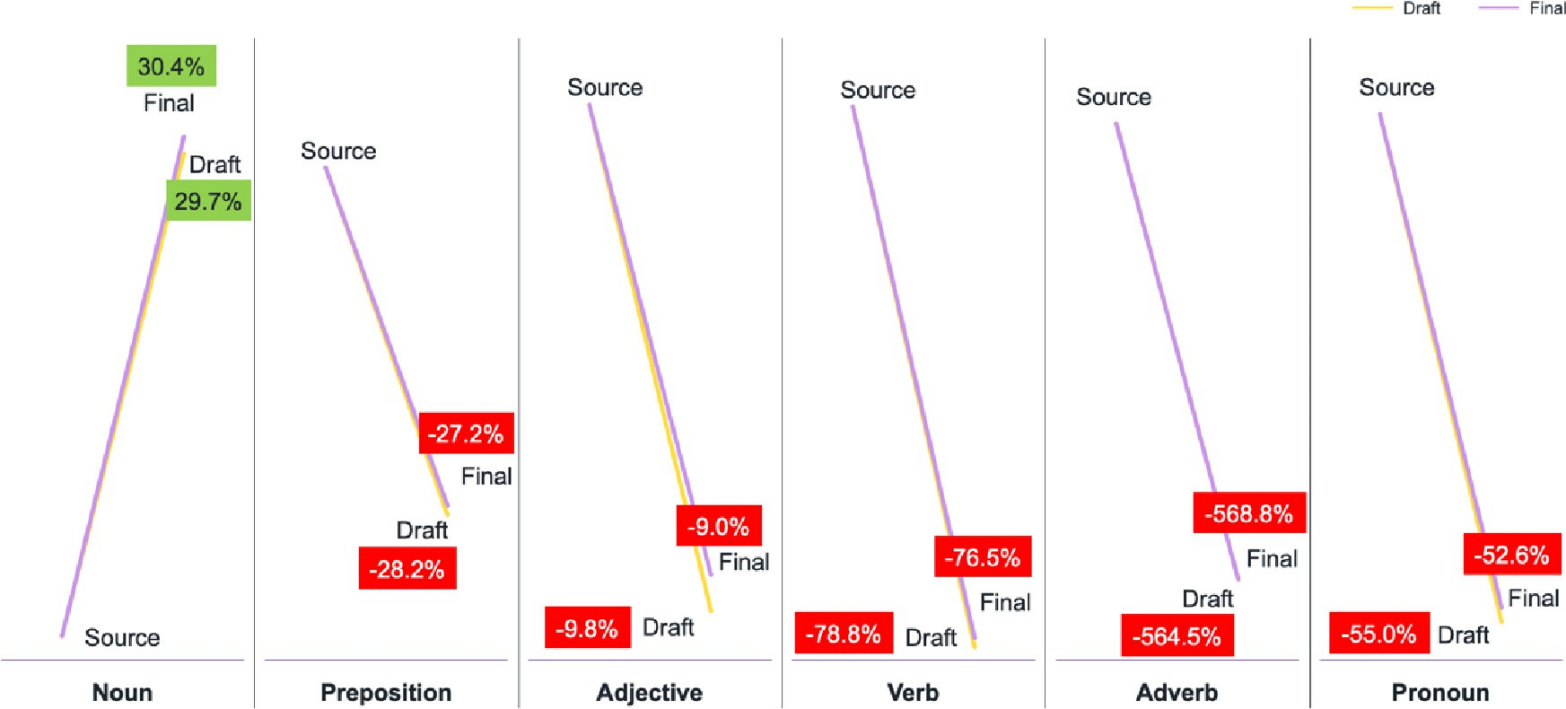
Changes in parts-of-speech averages across the SauLTC.

These numbers are further summarized in Fig 11, indicating a decrease in the POS across the board, barring nouns.

The differences in the POS distribution between English and Arabic may be attributed to the student translators’ tendencies in Arabic translation, such as the tendency to nominalize. Student translators may have chosen to translate some verbs as nouns, a class shift that can be used in translation for purely linguistic (e.g., when translating verbs of sense), stylistic, or pragmatic reasons. The numbers illustrate this class shift; on average, approximately 2,500 nouns and verbs were in each sub-corpus (see column titled “Total” in Table 4).

**Table 4:** Summation of the average number of nouns and verbs.

	Noun	Verb	Total
Source	1,327	1,057	2,384
Draft	1,887	591	2,478
Final	1,907	599	2,506

The other translation feature relates to complex verbs, such as “has been taken,” “go shopping,” “has become,” and “Where did she go?” English allows for a two-, three- or four-word verb phrase, which is impossible in Arabic. These examples were counted as two-word verbs and three-word verbs, but in Arabic, taggers regarded them as one-word verbs; for example, أخذ (“has been taken”), يتسوق (“go shopping”), and أصبح (“has become”). The POS count was measured in terms of the number of tokens, and it may posit English as being more verbose than Arabic in terms of verbs. Arabic is a pro-drop language; thus, in some cases, only one or two words are required when referring to something in the language, whereas three or four words are required in English. For example, the four-word question: “Where did she go?” corresponds to the two-word question أين ذهبت (“Where she went?”) in Arabic.

We must be careful not to generalize these findings because variables such as student translators’ GPAs can affect the results. However, a contrastive analysis of the lexico-grammatical characteristics of the two languages is not within the scope of this study.

A decrease in the number of pronouns can be explained by the use of implicit and appended pronouns in Arabic. Baker [25] noted that translators reduce ambiguity—a feature that can be reflected in the increased use of explicit references to individuals—to replace pronouns. More studies are recommended to investigate the pattern causing translated Arabic texts to have fewer pronouns than their corresponding English STs. Baker [25] argued that this phenomenon may indicate translators attempting to mediate the translated texts by making them less linguistically challenging. The uses of pronouns and adverbs within the corpus need a closer examination beyond this study’s capacity.

### 3.6 Corpus accessibility

There are two ways of accessing the SauLTC: first, the SauLTC website (www.saultc.net), which is currently in beta mode, is publicly available to the readers of this article at this stage of release. All types of search queries can be conducted on the website. Standard monolingual concordance lines can be produced, and parallelization in two side-by-side panes can be viewed on the website. Searches based on POS tagging are accessible through the pattern query page, and search queries can be controlled for variable interference through the participants’ metadata filters.

The second way to access the SauLTC is through the specially designed SauLTC application—a desktop download allowing users to search the corpus and edit the texts and profiles. It enables users to add additional learner translation folders. This application is only available to SauLTC researchers; however, a restricted version will be made available to SauLTC collaborators at other Saudi universities. There are also plans to upload the SauLTC to the corpus manager and text analysis software Sketch Engine [26]. This accessibility option will allow users to utilize Sketch Engine’s powerful text analysis tools to generate wordlists, extract keywords or terms, and calculate collocations and N-grams.

## 4. Discussion

### 4.1 Corpus uses

The SauLTC represents one of the first publicly available parallel LTCs for an English–Arabic language pair. Similar to other corpora, it can be utilized for pedagogical applications and research explorations.

#### 4.1.1 Pedagogical applications

All LTC initiatives emphasize the pedagogical benefits that students and instructors can gain from using these corpora in the classroom and for translator training in general, particularly if they incorporate error annotations [1]. Our corpus is a web-searchable resource specifically designed for didactic purposes. Several activities could be planned in the classroom using the corpus. One activity could be the detection of potentially problematic areas in the STs that need special attention from translators. The students must explain why they think some aspects of ST could be problematic and suggest translation procedures to solve them. Then, the students are asked to use the ST annotation scheme, which alerts them to potentially problematic areas in the ST. The students then compare their results with those of the scheme and reflect on the problems that were identified only by the scheme. Afterwards, they can check the information provided by the scheme relating to the problems identified. This exercise aims to sensitize students to the linguistic differences between the two languages that can create translation problems.

Students could also be asked to look up a specific English structure that is likely to be mistranslated (such as compound nouns or adjectives) and find out how students generally translate this structure. They examine concordance lines containing that structure and describe the different kinds of mistranslations identified. The next step is to consult the final versions for the correct translations. They can then discuss the various translation strategies that have been or could be employed to translate this structure. They can also compare their own initial translations and competency levels with those within the corpus. This activity can help students understand and characterize translation errors [27] and have a deeper insight into how and why they occur.

Another activity is to ask the students to reflect on the translation problems of a particular ST and edit it individually, in pairs, or groups. Then, they can compare their edited versions with the final translation versions in the corpus. This exercise makes them more aware of the areas where they need to improve their performance. This activity could also be used to implement peer assessment.

These activities could enhance the students’ autonomy and reflect aspects of the discovery learning approach proposed by Bernardini [28]. This approach champions open-ended, self-directed, learner-centered activities that encourage and lead students to be life-long learners and researchers. Additionally, such corpus-based activities develop students’ awareness, resourcefulness, reflective skills [29], and contrastive abilities.

Further, collecting and analyzing translation outputs can provide useful information for translation teaching and research. The learner translation characteristics can be utilized to develop learning and teaching materials and help teachers train student translators to establish equivalence, terminology, and phraseology between English and Arabic.

#### 4.1.2 Research explorations

The present corpus is designed to enable researchers to examine translation processes and products both quantitatively and qualitatively. For example, to investigate how the three sub-corpora exhibit linguistic patterns that are different in terms of lexical diversity and sentence length, we use different comparison measures. These calculations (see Table 5) in terms of the number of tokens and the number of types were performed using LancsBox software [30].

**Table 5:** SauLTC sentence and type/token statistics.

Sub-corpus	Sentence Length (mean)	Sentence Length (SD)	Word Length (mean)	Word Length (SD)	Types	Tokens	TTR	STTR	MATTR
Source	19.250	14.303	4.858	3.922	65169	1843885	0.035343	0.721166	0.721856
Draft	23.245	22.315	4.709	3.166	151402	1619661	0.093478	0.826296	0.82609
Final	23.789	23.351	4.713	2.4314	154826	1696840	0.091244	0.827462	0.827549

The first measure is the type-token ratio (TTR), a much-used measure of lexical diversity that measures the relationship between the number of different words in a corpus and the total number of words. Due to the TTR’s sensitivity to sample size, we use the STTR (standardized type-token ratio). The results indicate a lower ratio of the English source sub-corpus (0.72), indicating more repetition. By contrast, the Arabic draft and final students’ translation sub-corpora have a higher type-token ratio (0.83 for both versions), which indicates more significant lexical variation. This result reflects the diversity of the Arabic language due to it being a morphologically complex language.

Furthermore, the translators’ strategy of explicitation, e.g., the process of rendering information that is only implicit in the source text explicit in the target text [31, 32], plays a crucial role in text length. It is reasonable to assume that explicitation produces the effect that target texts are longer than the source text since it is generally achieved by adding explanatory words to the translation text. Therefore, in parallel text research, simplification and explicitation must be considered.

The second measure applied to compare the three sub-corpora is the average sentence length [31]. Table 5 illustrates standardized values for average sentence length and measures for the standard deviations of sentence length. The results demonstrate that the final submissions sub-corpus sentences appear to be the longest (23.8) and have the largest sentence length deviation. These observations need further examination to understand the variances in sentence lengths. Some of the issues that will be explored are why the Arabic translations tend to have larger deviations in sentence lengths. We will examine if there are any significant differences among individual translation students. Other reasons for these variances might be the Arabic language, translation, or editing. In this ongoing study, we attempt to weigh the effect of each of these reasons while exploring others.

Paradoxically, unlike sentences, texts are shortened when they are translated from English into Arabic. A comparison of text length between the source text and the translated texts illustrates that the English source texts contain 1,843,885 tokens while the Arabic final translations have 1,696,840 tokens, i.e., the translated Arabic text is approximately 147,045 tokens (8%) less than the English source text. A relatively similar picture is to be obtained if one compares the English source text with the draft translated texts, in which the translations consist of 1,619,661 tokens, approximately a 12% lower text length than the source text. According to translational studies, one of the leading translation strategies is simplifying the translated text. Empirically, this can be seen at the lexical level, where a smaller number of tokens of the target texts can be understood as a lexical simplification [33]. Another rationale for the differences obtained in text length might not be caused by the stylistic preferences of the translators or by an explicitation of the original English text but by Arabic’s morphological or morphosyntactical features. Further investigation is ongoing to explore whether the relationship between translation and text length is affected by the morphological and syntactic differences between the two languages under examination.

SauLTC could potentially provide researchers with opportunities to explore novel ideas and research areas, such as comparing the areas of difficulty in different genres and associated errors in each genre. It is customary in linguistic research to examine students’ errors to predict difficulty areas for learners. We could identify areas of difficulty, then explore the different types and levels or severity of errors associated with these areas. This kind of research can have implications for translation course design as it informs teachers’ choice of texts.

Translation features (such as explicitation, normalization, and simplification) are another area that could be investigated in LTC, especially in non-European languages [34]. According to Granger and Lefer [1], this area is a much-needed endeavor as exploring these features in LTCs is still preliminary compared to research done on professional translation. Students’ translations could be compared to original Arabic texts to determine how far these features are characteristic of their translated outputs. Another research question is to see if these students adhere to typical Arabic language conventions, for example, or if the ST patterns constrain their translations. Another area that needs more attention is the differences between learner and professional translations [1]. Different linguistic phenomena in the relevant two types of corpora can be examined and compared to better characterize learner translations. Kunilovskaya, Morgoun, and Pariy [35] suggested that quantitative tendencies in students’ translations that do not align with professional translation and non-translation could be examined. This can help teachers provide useful guidance to their students to enhance the naturalness and fluency of their translation output.

The SauLTC is one of the first corpora to include parallel pre- and post-editing versions of trainee translations. Researchers can explore the translation process using numerical data and statistical measures. They can assess and measure various aspects of the translation, such as the frequency of specific words or phrases, the distribution of translation errors, or lexical complexity [36, 37]. Also, researchers can study the translation process by assessing the translations’ quality, accuracy, and linguistic features. They can analyze the translators’ choices and evaluate their appropriateness in capturing the meaning and intent of the original text. Through quantitative and qualitative analyses, the corpus provides researchers with a comprehensive toolkit to explore and gain insights into the translation process. They can examine the numerical and subjective dimensions of translation, leading to a more holistic understanding of how texts are transformed from English to Arabic.

The corpus is a valuable resource for the automatic processing of bilingual texts. Because it is aligned at the sentence level, it can be used to automatically predict English–Arabic word alignments and inform lexical and semantic dictionaries. The SauLTC also has many potential applications and uses for training translation models, evaluating translation quality, and investigating specific linguistic phenomena in learner translations.

Another recommendation is the examination of the interdependence of several variables. SauLTC provides rich metadata for the three types of participants involved. The metadata includes not only the student translators’ ages, GPAs, assignment grades, and the instructors’ and alignment verifiers’ backgrounds. The differences among student translators and the effectiveness of instructor feedback on their production are two potential areas for exploration. Moreover, the instructors’ and assistants’ participation in the assessment and sentence alignments can be analyzed for their overall and inter-individual variation effects. The SauLTC XML Tool, developed to extract tables and figures, will allow researchers to investigate the translation of such extra-textual elements.

Additionally, the documentation of the feedback provided by the verifiers, who double-checked the automatic sentence alignment, allows for an additional avenue of investigation. The effectiveness of the alignment software, the influence of the genre on the accuracy, and inter-individual variation among the verifiers are some areas that may be examined. Overall, the possible applications of an English–Arabic LTC are numerous and valuable for research, training, and pedagogical purposes.

Finally, researchers can assess the impact of using SauLTC on the development of students’ translation competence and all its sub-competences such as (using PACTE’s model terminology [38]), knowledge about translation sub-competence, strategic sub-competence, instrumental sub-competence, and bilingual competence.

## 5. Conclusion

This article presented some of the tools developed specifically for the corpus. First, the SauLTC XML Conversion Tool was designed to convert MS Word files into an XML standard format and to extract tables and figures to demonstrate if and how these extra-textual elements are translated. Second, we introduced the SauLTC desktop application, an automatic extraction tool developed to store student translators’ productions, tackle the alignment of Excel sheets, and query the corpus offline. This tool facilitates the project’s expansion to other universities, as we plan to partner with several Saudi universities on this national-level project. These ideas for improvements and expansion meet the needs of language learners, translators, and researchers for future research. Like most works in the field, our corpus focuses on translations into student translators’ native languages to research L1 translation-related phenomena and inform language pedagogy. This resource provides a foundation for investigating various issues related to the distinctive nature of translated texts, the style of translation learners, the impact of English as the source language on the patterning of Arabic, the effect of the text genre on translation strategies, and other issues of interest to both translation scholars and linguists. Most importantly, this corpus was designed to aid teachers in using concordances for class preparation by helping them target specific difficulties and incorporate error analysis as an exercise for student translators to enhance their critical reading and translation assessment skills. Using the corpus in the classroom offers learners self-evaluation and cooperative learning opportunities and motivates their self-learning engagement.
